# Right ureterovesical junction cyst associated with ipsilateral renal agenesis: a case report of a possible Wolffian duct maldevelopment

**DOI:** 10.3389/fmed.2026.1766840

**Published:** 2026-02-24

**Authors:** Junpeng Liu, Xinyao Sun, Jiaxi He, Zeke Zhang, Derong Zhou, Bin Huang, Jinhuan Yang, Yutong Fang, Hao Lin

**Affiliations:** 1 Department of Urology, The Second Affiliated Hospital of Shantou University Medical College, Shantou, China; 2 Department of Urology, The First Affiliated Hospital of Sun Yat-sen University, Guangzhou, China; 3 Department of Breast Surgery, Cancer Hospital of Shantou University Medical College, Shantou, Guangdong, China

**Keywords:** case report, congenital malformation, ureterovesical junction cyst, Wolffian duct anomaly, Zinner syndrome

## Abstract

**Introduction:**

Right ureterovesical junction cyst associated with ipsilateral renal agenesis is an extremely rare congenital anomaly of the male genitourinary tract, likely resulting from abnormal development of the Wolffian duct and ureteric bud. We report the case of a 35-year-old man with a one-year history of progressive dysuria. Contrast-enhanced computed tomography (CT) revealed right renal agenesis and cystic dilatation of the intramural segment of the right ureter protruding into the bladder cavity, with the proximal ureter showing tortuosity and atresia. The contralateral kidney and ureter were normal. The patient underwent laparoscopic right ureterectomy combined with transurethral bladder exploration and left ureteral stent placement. Histopathological examination confirmed a ureterovesical junction cyst with chronic inflammation and cystic wall change. The postoperative course was uneventful.

**Conclusion:**

This case highlights a rare Wolffian duct–related developmental anomaly distinct from classical Zinner syndrome, emphasizing the importance of recognizing atypical presentations for accurate diagnosis and optimal surgical management.

## Introduction

Congenital anomalies of the Wolffian duct system are uncommon and may involve the kidney, ureter, and seminal vesicle. Zinner syndrome, characterized by the triad of ipsilateral renal agenesis, seminal vesicle cyst, and ejaculatory duct obstruction, is extremely rare (incidence ≈0.0046% in males) (1). These anomalies result from developmental defects of the mesonephric duct and ureteric bud during early embryogenesis (2).

Most reported Wolffian duct anomalies involve seminal vesicle cysts or ejaculatory duct obstruction, whereas isolated cystic dilatation of the distal ureter or ureterovesical junction associated with renal agenesis is exceptionally rare (3). A literature search identified only a single case report published in 2017, highlighting the scarcity of documented cases and making it difficult to estimate the precise incidence of this condition (4). Such cases may mimic Zinner syndrome on imaging but differ in anatomic origin and pathogenesis.

The present report describes a rare case of right ureterovesical junction cyst associated with ipsilateral renal agenesis, which presented with progressive dysuria due to bladder outlet obstruction. This case highlights the importance of careful differentiation from classical or variant forms of Zinner syndrome and provides insight into the embryological basis, imaging features, and surgical management of this unusual Wolffian duct–related anomaly.

## Case report

A 35-year-old Chinese man presented with a one-year history of progressive dysuria characterized by urinary stream thinning. He denied fever, hematuria, or flank pain. His past medical history included well-controlled hypertension and gout; there was no family history of genitourinary disorders. Physical examination and laboratory findings were unremarkable.

**Figure 1:**
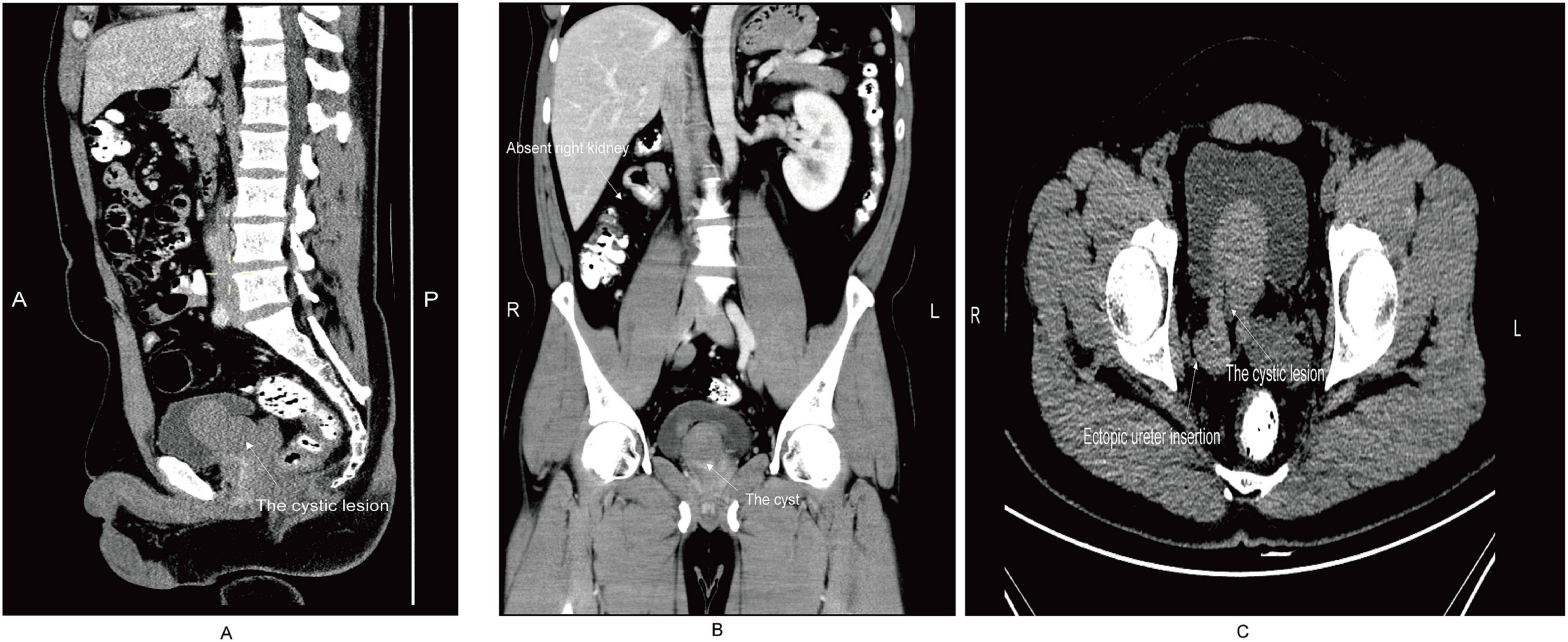
Contrast-enhanced computed tomography (CT) findings of the patient. **(A)** Sagittal CT image demonstrating a cystic lesion arising from the right seminal vesicle, protruding into the bladder cavity (arrow). **(B)** Coronal CT image showing right renal agenesis and a well-defined cystic mass located posterior to the bladder, consistent with a seminal vesicle cyst (arrow). **(C)** Axial non–contrast-enhanced axial CT image revealing an ectopic insertion of the right ureter into the right seminal vesicle, with the cyst partially protruding into the bladder lumen (arrows).

Contrast-enhanced computed tomography (CT) revealed right renal agenesis with a residual right ureter, showing proximal atresia and an abnormal distal ureteral course, raising suspicion of communication with the right seminal vesicle. The right seminal vesicle appeared cystically dilated on imaging, forming a cystic lesion that protruded into the bladder cavity (Figures 1A–C). The overall radiological impression suggested right renal agenesis with suspected distal ureteral communication to the vesicle and a vesicle cyst protruding into the bladder, mimicking the imaging features of Zinner syndrome. The vesicle cyst appeared as a well-defined, unilocular, low-attenuation lesion (approximately 16 HU) with a thin and smooth wall, without internal septations or solid components. The residual distal ureter communicated directly with the cyst, but the proximal ureteral segment was not visualized, reflecting atresia. These imaging features could potentially lead to misdiagnosis as a bladder cyst or prostatic cyst if the anatomical relationship with the ureter and seminal vesicle is not carefully evaluated.

**Figure 2:**
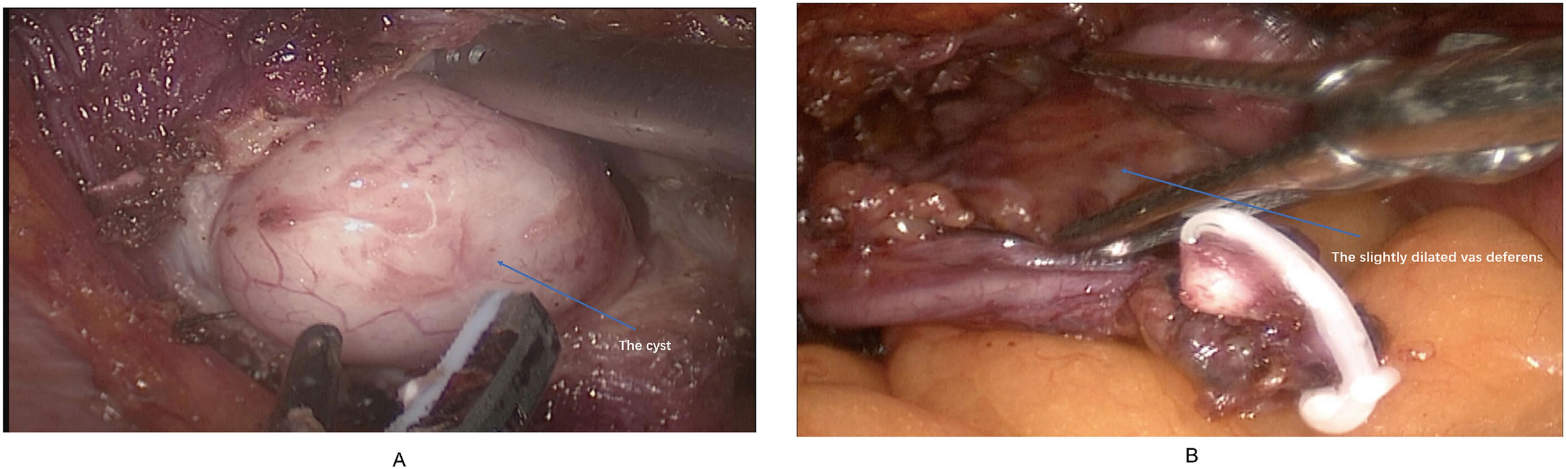
**(A)** Intraoperatively, a cystic dilatation was observed in the intramural segment of the right ureter. **(B)** The vas deferens near the seminal vesicle showed mild dilatation without cyst formation.

The patient underwent transurethral bladder exploration and laparoscopic right ureterectomy combined with right vasectomy under general anesthesia. After successful establishment of pneumoperitoneum and trocar placement, the right ureter was identified and dissected along its course to the bladder wall. Contrary to the preoperative imaging findings, a cystic lesion approximately 5 cm in diameter was identified within the distal intramural segment of the right ureter near the bladder neck, containing yellowish-white purulent fluid (Figure 2A). The right seminal vesicle appeared normal, and no communication was observed between the cystic lesion and the seminal vesicle. The vas deferens near the seminal vesicle was slightly dilated (Figure 2B). The dilated distal ureteral segment and the right vas deferens were excised completely. The bladder wall was closed with continuous absorbable sutures, and a pelvic drain was placed. The operation was uneventful, with minimal blood loss (approximately 10 mL), and the patient recovered smoothly from anesthesia.

**Figure 3:**
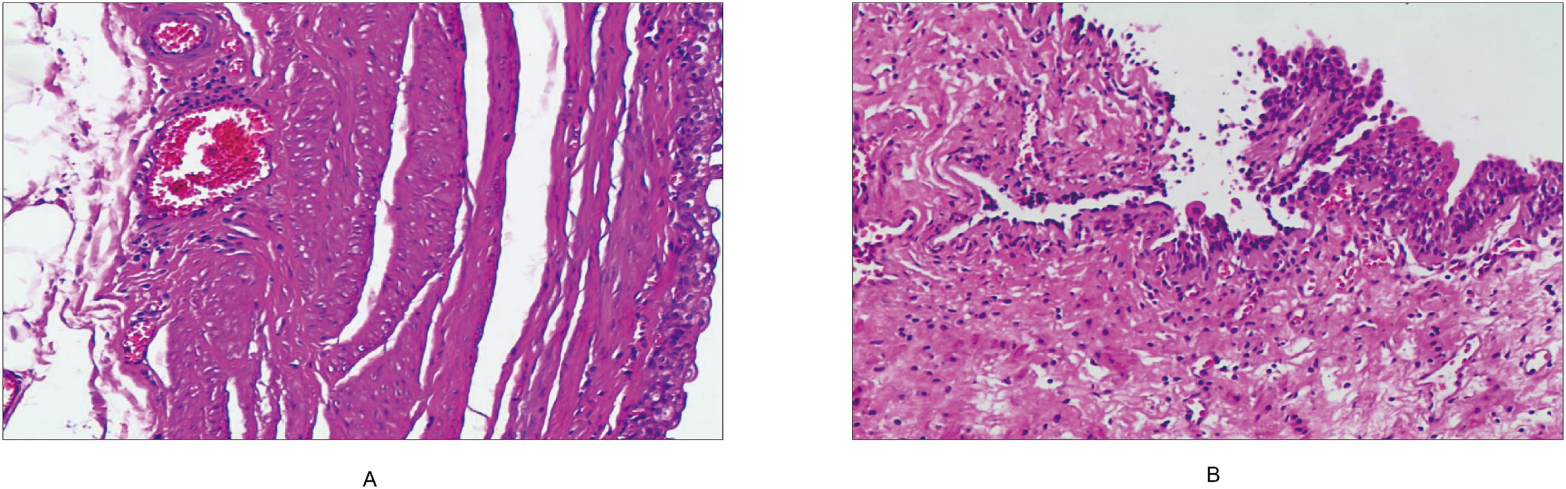
**(A)** The right ureter exhibits cystic dilatation with chronic inflammatory changes. **(B)** Benign vas deferens tissue is observed.

Histopathological examination showed cystic dilatation of the ureter with chronic inflammation and urothelial lining (Figure 3A), confirming a ureteral cyst. Benign vas deferens tissue was also observed (Figure 3B). At one-month follow-up, the patient’s dysuria had completely resolved without recurrence.

## Discussion

This case represents a rare Wolffian duct–related anomaly where ipsilateral renal agenesis coexisted with cystic dilatation confined to the distal ureter. Although imaging suggested Zinner syndrome, intraoperative and pathological findings confirmed a ureteral remnant cyst.

Zinner syndrome results from abnormal development of the mesonephric duct and ureteric bud, producing renal agenesis with ipsilateral seminal vesicle cyst and ejaculatory duct obstruction (5). Our case shared renal agenesis but differed in several key aspects: (1) a normal seminal vesicle with no communication to the cyst; (2) cystic dilatation restricted to the ureteral wall with purulent content; and (3) histology showing urothelium-lined cyst consistent with a ureterovesical junction cyst. In addition, it differed in (4) clinical presentation, with urinary tract–related symptoms rather than reproductive or ejaculatory complaints; (5) cyst content and epithelial lining, with purulent material and urothelial lining instead of the clear or mucinous fluid and glandular/cuboidal epithelium typical of seminal vesicle cysts; and (6) embryologic origin, representing a Wolffian duct–derived ureteral remnant rather than a true seminal vesicle malformation. These distinctions indicate a Wolffian duct–derived ureteral remnant cyst rather than true Zinner syndrome, though both share a common embryologic origin (6, 7). Recognition of this variant is essential for accurate diagnosis and surgical management of similar bladder-outlet lesions. Although the patient did not undergo magnetic resonance imaging (MRI), previous studies suggest that MRI may provide superior soft tissue contrast and better delineation of the relationship between the ureteral remnant, seminal vesicle, and bladder (8). T1- and T2-weighted sequences can help characterize cyst contents, differentiate purulent material from mucinous or serous fluid, and potentially improve preoperative diagnostic accuracy.

The embryologic mechanism of this condition likely involves abnormal development of both the Wolffian duct and the ureteric bud (9). Normally, the ureteric bud arises from the mesonephric duct and induces formation of the metanephric kidney, while the distal Wolffian duct contributes to the formation of the seminal vesicle and ejaculatory duct (10). In our case, renal agenesis suggests early failure of ureteric bud induction or interaction with the metanephric blastema. The cystic dilatation confined to the distal ureter may reflect localized persistence of the Wolffian duct segment that failed to regress or canalize properly, resulting in a ureteral remnant cyst (11). This differs from Zinner syndrome, in which cystic changes involve the seminal vesicle due to obstruction of the ejaculatory duct, despite sharing a common embryologic origin (12). This proposed pathogenic link provides a mechanistic explanation for the unique anatomical and histological features observed in this patient.

Although limited to a single observation, this case expands the clinical spectrum of Wolffian duct–related anomalies and underscores the importance of correlating imaging findings with intraoperative anatomy and histopathology.

### Patient perspective

The patient’s symptoms of dysuria have been significantly improved.

## Data Availability

The raw data supporting the conclusions of this article will be made available by the authors, without undue reservation.

## References

[1] Hergan B Fellner FA Akbari K. Incidental imaging findings suggesting Zinner syndrome in a young patient with pulmonary embolism: a case report. Radiol Case Rep. (2020) 15:437–41. doi: 10.1016/j.radcr.2020.01.027, 32148603 PMC7033301

[2] Karki P Manandhar S Kharel A. A rare case of Zinner syndrome: triad of unilateral renal agenesis, ipsilateral seminal vesicle cyst and ejaculatory duct obstruction. Radiol Case Rep. (2021) 16:3380–2. doi: 10.1016/j.radcr.2021.08.012, 34504629 PMC8411213

[3] Gupta V Khan RK Kumar LP. Zinner syndrome: a mesonephric duct anomaly with renal agenesis, ipsilateral seminal vesicle cyst, and ejaculatory duct obstruction. Med J Armed Forces India. (2024) 80:S238–s242. doi: 10.1016/j.mjafi.2022.05.012, 39734907 PMC11670647

[4] Ahmed A. Ipsilateral renal agenesis with megaureter, blind end proximal ureter and ureterocele in an adult. J Ayub Med Coll Abbottabad. (2017) 29:150–3. 28712197

[5] Deen S Arora A Lunawat R. Zinner syndrome: ipsilateral renal agenesis and seminal vesicle cyst presenting with bony metastasis. Cureus. (2022) 14:e28949. doi: 10.7759/cureus.28949, 36237803 PMC9547664

[6] Kwenda EP Locke RA Archer JS Su LM Shenoy A DeMarco RT . Robot-assisted laparoscopic resection of the mesonephric duct remnant in a patient with Zinner syndrome. J Endourol Case Rep. (2020) 6:198–201. doi: 10.1089/cren.2020.0020, 33102726 PMC7580635

[7] Campora M Olivero A Toncini C Spina B Fulcheri E Terrone C . Zinner syndrome: a diagnostic challenge. The aid of morphology, embryology, and immunohistochemistry. Urology. (2017) 108:e3–5. doi: 10.1016/j.urology.2017.06.013, 28684259

[8] Bas D Nalbant MO. Zinner syndrome: radiologic diagnosis in a rare case. Curr Med Imaging. (2023) 2023:133. doi: 10.2174/1573405620666230829150133, 37649289

[9] Shah S Patel R Sinha R Harris M. Zinner syndrome: an unusual cause of bladder outflow obstruction. BJR Case Rep. (2017) 3:20160094. doi: 10.1259/bjrcr.20160094, 30363237 PMC6159234

[10] Kummari S Ranga M. Zinner syndrome in a young male: a case report and review of the literature. Cureus. (2024) 16:e74909. doi: 10.7759/cureus.74909, 39742185 PMC11687404

[11] Darcy DG Yao-Cohen M Olson TR Downie SA. Unilateral complete agenesis of mesonephric duct derivatives in an 82-year-old male cadaver: embryology, anatomy and clinical considerations. Urol Case Rep. (2017) 15:20–2. doi: 10.1016/j.eucr.2017.06.003, 28879095 PMC5582374

[12] Gorantla R Allu S Rao A. A triad of unilateral renal dysgenesis with ipsilateral seminal vesical and ejaculatory duct obstruction: an uncommon urogenital congenital anomaly, Zinner syndrome-a case report. Indian J Radiol Imaging. (2021) 31:707–9. doi: 10.1055/s-0041-1735503, 34790319 PMC8590544

